# Self-Resolving Pulsatile Frontal Mass Following Blunt Head Trauma

**DOI:** 10.1016/j.acepjo.2024.100025

**Published:** 2025-01-13

**Authors:** Yoshihiro Aoki, Koichi Hayakawa, Kazuhiko Suyama

**Affiliations:** 1 Coordination Office for Emergency Medicine and International Response, Acute and Critical Care Center, Nagasaki University Hospital, Nagasaki, Japan; 2 Department of Emergency Medicine, Nagasaki Harbor Medical Center, Nagasaki, Japan; 3 Department of Neurosurgery, Nagasaki Harbor Medical Center, Nagasaki, Japan

**Keywords:** computed tomography angiography, head injury, pseudoaneurysm, superficial temporal artery, trauma

## 1 Case Presentation

A 58-year-old man was hospitalized following a traffic accident involving a light truck. He arrived febrile, SARS-CoV-2 antigen positive, and conscious without neurologic signs. Computed tomography of the head revealed traumatic subarachnoid hemorrhage, basilar skull fracture with pneumocephalus, left periorbital and zygomatic fractures, and multiple left rib fractures. Contrast-enhanced computed tomography showed a right frontal subcutaneous hemorrhage and a 9 mm pseudoaneurysm in the periphery of the frontal branch of the right superficial temporal artery (STA) without an adjacent convexity fracture (Fig A,B). He was admitted for blood pressure regulation, pain management, cerebral monitoring, and rehabilitation.

**Figure:**
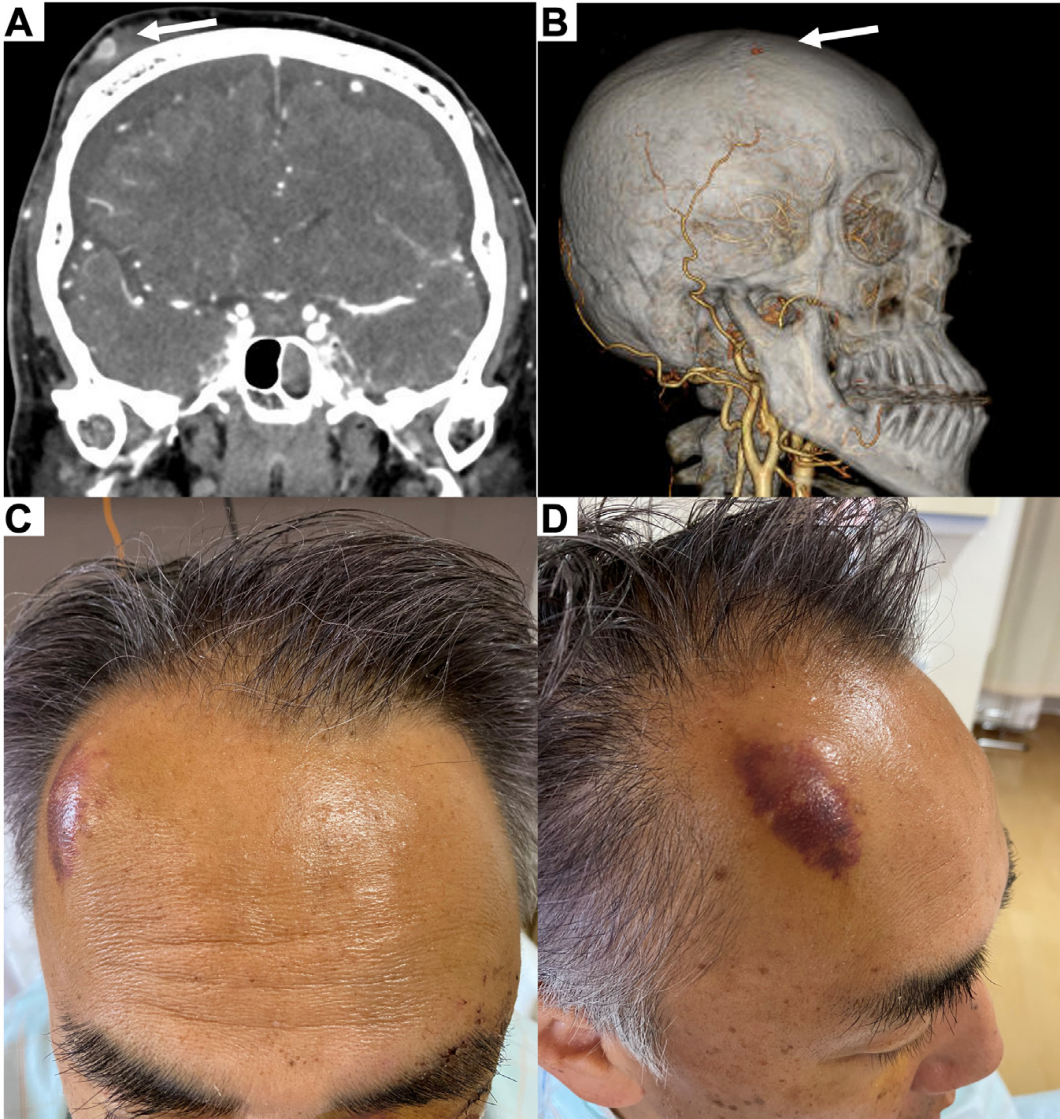
A, Contrast-enhanced computed tomography scan revealing a right frontal subcutaneous hemorrhage with a 9 mm pseudoaneurysm. B, Computed tomography angiography demonstrating a 9 mm pseudoaneurysm at the periphery of the frontal branch of the right superficial temporal artery. C and D, Clinical image of a pulsatile mass that appeared in the right frontal area on the day following admission.

## 2 Diagnosis: Traumatic Superficial Temporal Artery Pseudoaneurysm

The next day, a pulsatile mass appeared in the right frontal area (Fig C,D), not necessitating surgical intervention during the hospital stay. He was discharged on day 17, and his 2-week follow-up showed that the mass was pulseless and had resolved without surgery. Although the spontaneous resolution of a traumatic STA pseudoaneurysm has been reported following long-term observation, the gold standard is still considered to be surgical or radiologic intervention.^1^, ^2^, ^3^, ^4^ A rapidly developing closed-traumatic pseudoaneurysm in the periphery of the STA can resolve spontaneously, as demonstrated in our case.

## Funding and Support

By *JACEP* *Open* policy, all authors are required to disclose any and all commercial, financial, and other relationships in any way related to the subject of this article as per ICMJE conflict of interest guidelines (see www.icmje.org). The authors have stated that no such relationships exist.

## Conflict of Interest

All authors have affirmed they have no conflicts of interest to declare.
